# Non-invasive single slice estimate of aortic distensibility from phase-contrast MRI: application to hypoplastic left heart syndrome

**DOI:** 10.1186/1532-429X-14-S1-P117

**Published:** 2012-02-01

**Authors:** Giovanni Biglino, Jennifer A Steeden, Catriona Baker, Silvia Schievano, Tain-Yen Hsia, Alessandro Giardini, Andrew M Taylor

**Affiliations:** 1 Centre for Cardiovascular Imaging, UCL Institute of Cardiovascular Science, London, UK; 2 Cardiorespiratory Unit, Great Ormond Street Hospital for Children, London, UK

## Background

Knowledge of aortic distensibility (D) is important in several congenital scenarios. For instance, palliation of hypoplastic left heart syndrome (HLHS) often involves surgical arch reconstruction with a patch. Patch calcification is likely to impinge on aortic arch D, with a detrimental effect on ventriculo-arterial coupling. D can thus be a useful measure to derive. In order to estimate it, pressure change data from invasive measurements is combined with area (A) change information. Alternatively, distensibility can be derived from wave speed. This study proposes a method for calculation of wave speed from a single phase-contrast MRI measurement and consequently single-point estimate of D, applying the method to a cohort of HLHS patients.

## Methods

**Figure 1:**
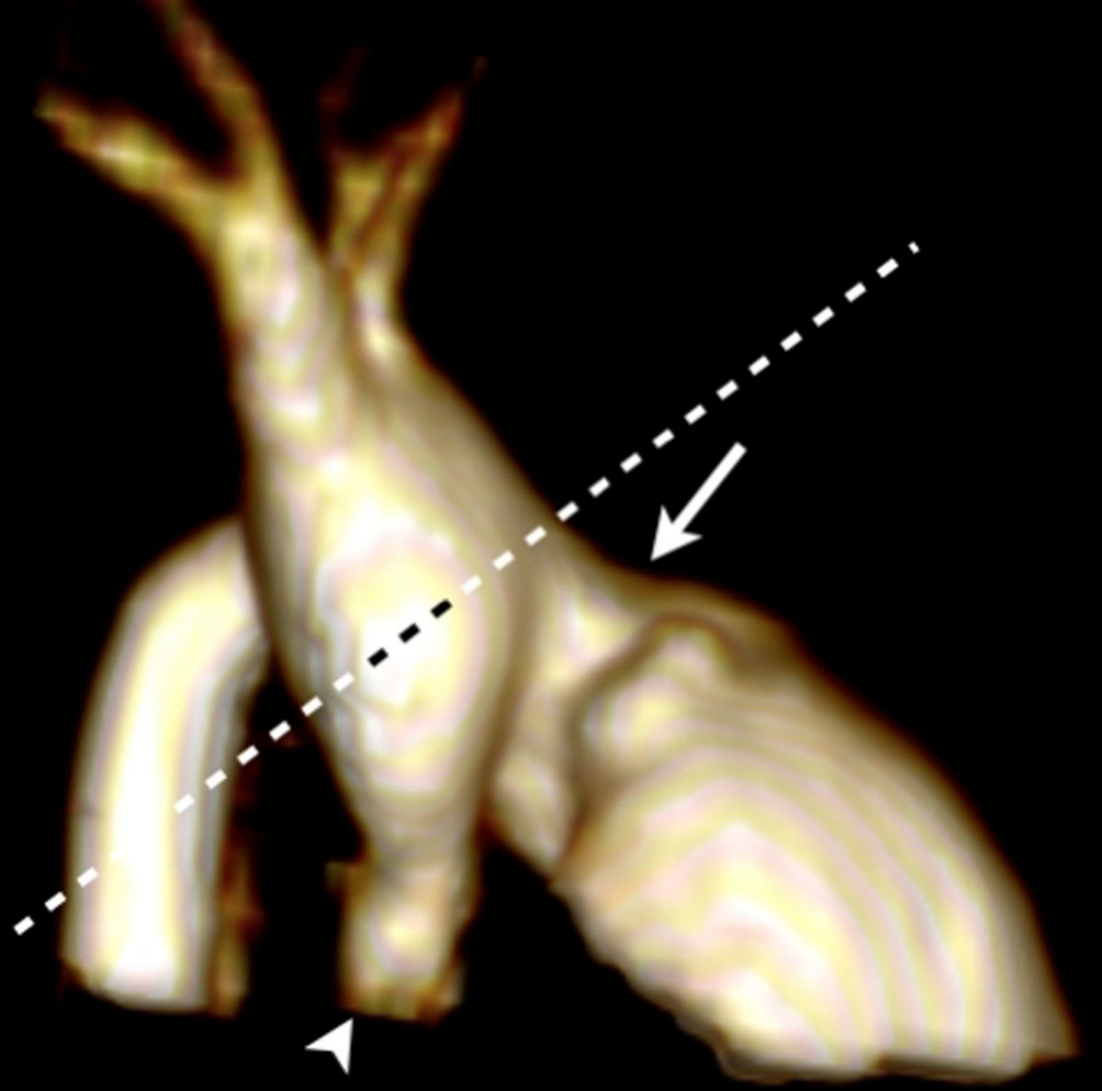
Position of the imaging plane above the Damus-Kaye-Stansel, capturing the reconstructed ascending aorta. Arrow - neo-aorta, arrowhead - native hypo plastic aorta.

Ten patients with HLHS and aortic arch reconstruction (3.4±1.0 years, BSA = 0.6±0.1 m2) were assessed with MRI pre-total cavil pulmonary connection (TCPC) completion and compared with another ten, non-HLHS patients (4.7±1.5 years, BSA = 0.7±0.1 m2) also imaged pre-TCPC. Their ascending aortic flow sequence was analysed with an in-house written plugin (Osirix). Care was taken that, for the HLHS group, the flow sequence was acquired after the Damus-Kaye-Stansel, ensuring that the reconstructed portion of the arch was captured (Figure 1). Area (A) and velocity (V) were extracted with a previously validated automatic propagation algorithm based on nonrigid registration. These data were interpolated and smoothed. Then, a loop was obtained plotting lnA versus V. The fractional change in A and changes in V are related by the waterhammer equation, dV=cdlnA, where c=wave speed. The slope of the linear part of the loop (early systole) thus yields c. D can finally be derived using the Bramwell-Hill equation, c2=1/ρD, where ρ=blood density.

**Figure 2:**
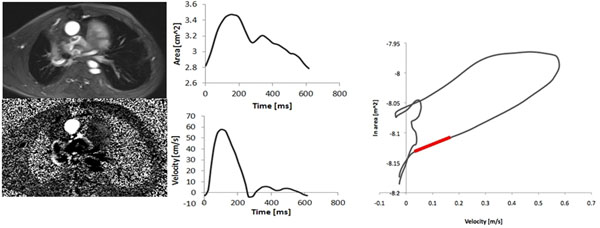
Magnitude and phase-contrast images (left), from which area and velocity signals are derived (centre). Velocity and ln(area) information are combined in order to obtain a loop (right), whose initial slope yields the value of wave speed (red line), directly related to distensibility.

## Results

HLHS patients showed significantly higher wave speed (8.4±2.4 vs 4.5±0.9 m/s, p=0.00013) and, in turn, reduced aortic D due to the reconstructed arch (0.0023±0.0015 vs 0.0068±0.0029 1/mmHg, p=0.00075). Concomitantly, the HLHS cohort also had reduced cardiac output (3.3±0.6 vs 4.8±2.0 l/min, p=0.046) and ejection fraction (52.2±6.3 vs 60.1±4.7 %, p=0.005). This clearly suggests a link between the changes in aortic D following surgery and the coupling between the single ventricle and the artery, as the variation in D results in change in vascular impedance.

## Conclusions

This work presents a method for local estimation of distensibility based on wave speed derived from one phase-contrast measurement, without the necessity of acquiring multiple slices for foot-to-foot estimate. Application of this method to a HLHS population has shown the detrimental effect of surgical arch reconstruction on distensibility.

## Funding

Fondation Leducq, Royal Academy of Engineering, EPSRC, UK National Institute of Health Research (NIHR).

